# Supplementary material for: A bvrR/bvrS Non-Polar Brucella abortus Mutant Confirms the Role of the Two-Component System BvrR/BvrS in Virulence and Membrane Integrity
Source: Microorganisms. 2023 Aug 5;11(8):2014. doi: 10.3390/microorganisms11082014 (PMC10459465; doi:10.3390/microorganisms11082014)
Supplement: Supplementary file 1 [file microorganisms-11-02014-s001.zip › microorganisms-2503526-supplementary.pdf]

microorganisms

MDPI

Supplementary Material

Table S1. List of primers used in this study

Primer	*Sequence*
bvrRS-Up-For	GCGGATCCAGCACGATCTTGCGGGTAAGA
bvrRS-Up-Rev	CTTCATGCGGACCTTCTCCTTT
bvrRS-Dn-For	GCATGACCGTGAAAAGTGGATATC
bvrRS-Dn-Rev	GCCTGCAGCATCACGGAAATGGCCAGATT
bvrRS-Con-For	CGTTTAACCCGAGCGTTCTGG
bvrRS-Con-Rev	CATTTGCCGACAATGCCGG
bvrR_bv1-For	CATGGACGGTATGGAGCTTCTG
bvrR_bv1-Rev	GAGGAAGATGACCGGCAGATC
bvrRS-South-For	ACCACCCTGCAATTGCTGG
bvrRS-South-Rev	ATATCGCTCAGCCTCGTCGC
pckA-For	GCACGACATTTTCCAGAACC
pckA-Rev	TGTGGTGGGACAACAACAAG

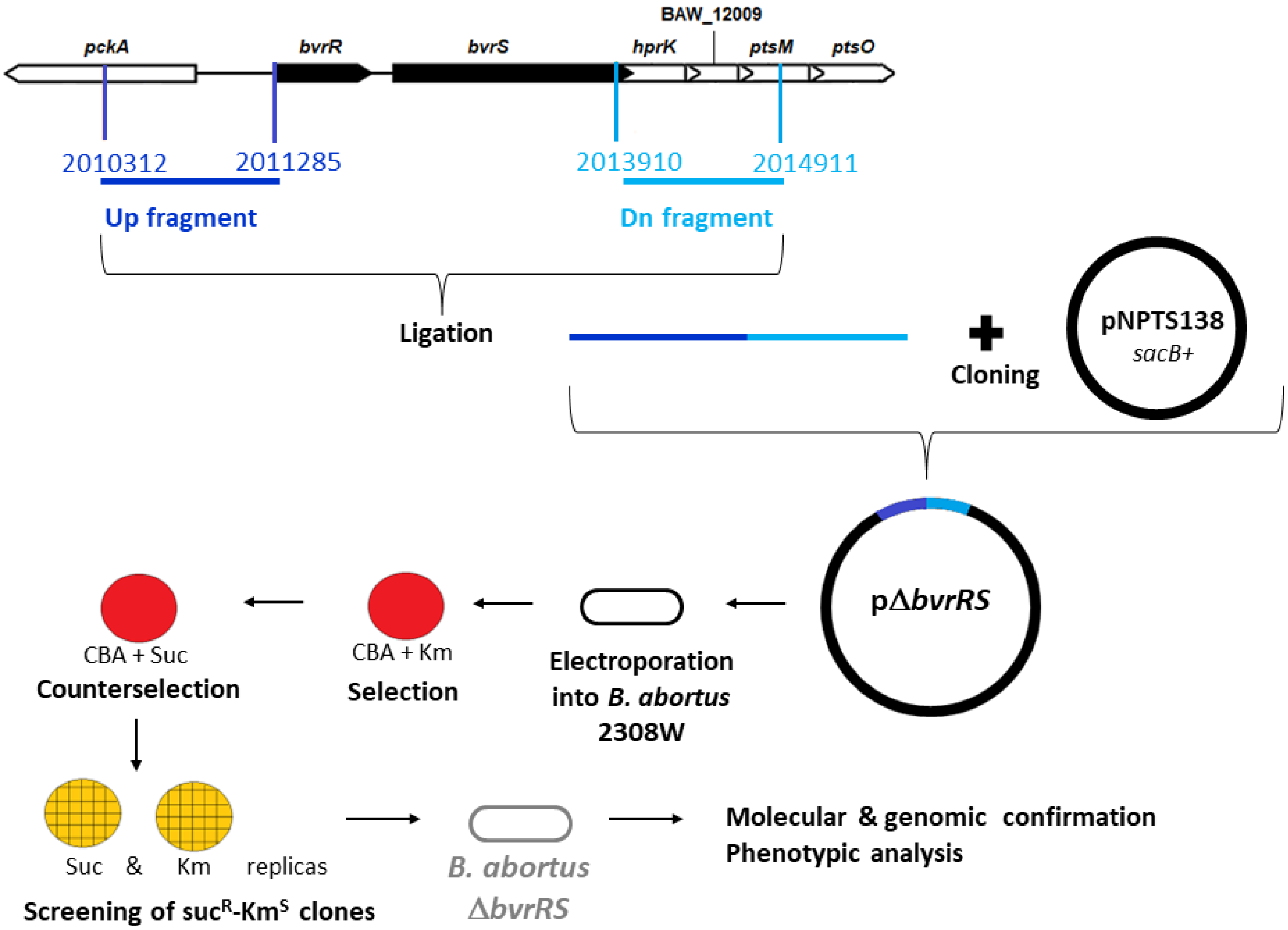

Figure S1. Schematic representation of the unmarked gene excision strategy used in this study for the construction of the mutant strain *B. abortus* ΔbvrRS. The Up and Dn fragments were amplified, ligated and cloned into pNPTS138, a suicide vector with a kanamycin (km) resistant marker and the gene *sacB* for sucrose (*suc*) counterselection. The resulting plasmid was called pΔbvrRS and was electroporated into the parental strain *B. abortus* 2308W. During the counterselection, the construct was integrated into the bacterial chromosome by allelic exchange, resulting in a deletion of *bvrR*/*bvrS* coding sequences and plasmid loose. The suc^R and Km^S clones were screened by PCR and the mutation was further confirmed by Southern Blot, DNA Sanger sequencing and Western Blot. Biochemical identification as *Brucella* sp., was also confirmed, and the phenotype of the mutant strain *B. abortus* ΔbvrRS was analyzed and compared to the phenotype of both transposition mutants.

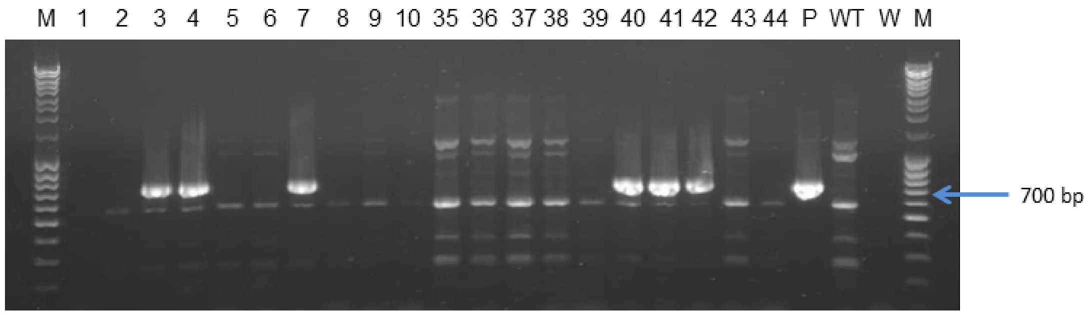

(a)

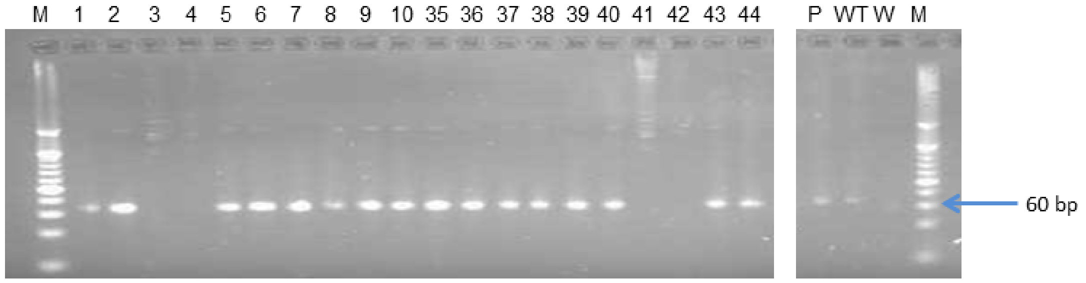

(b)

Clones	Con amplicon	bv1 amplicon	Interpretation
3, 4, 40, 41	+	-	Mutant
7, 40	+	+	Merodiploid
1, 2, 5, 6, 8, 9, 10, 35, 36, 37, 38, 39, 43, 44	-	+	Revertant

(c)

Figure S2. PCR screening of Suc^R y Km^S clones. (a) PCR results obtained in a reaction with bvrRS-Con primers (Supplementary Table 1), amplifying a 709 bp fragment in the ΔbvrRS mutant strain; (b) PCR results obtained in a reaction with bvrR_bv1 primers (Supplementary Table 1), amplifying a 63 bp internal fragment of *bvrR* coding sequence not expected to be amplified in the in the ΔbvrRS strain. M= Molecular marker MassRuler DNA Ladder Mix, ready-to-use (Thermo Fisher Scientific, USA) or O'RangeRuler 20 bp DNA Ladder, ready-to-use (Thermo Fisher Scientific, USA); P=plasmid pΔbvrRS; WT= Wild Type parental strain *B. abortus* 2308W; W= water; (c) Interpretation of the PCR screening. The clones 3, 4, 40 and 41 were considered to harbor the double deletion of the *bvrR*/*bvrS* genes. The clones 7 and 40 were considered merodiploids and the rest of the clones tested were considered revertant. The results are representative of at least three independent experiments.

Table S2. Quality results of *B. abortus* sequencing and whole genome assembly

Metric	*Brucella abortus 2308W*	*Brucella abortus ΔbvrRS*
Total no. of reads	24,882.0	48,431.0
Mean read length	8,892.1	8,101.2
Mean read quality	13.6	13.6
Sequencing depth (×)	68	121
Genome size (bp)	3,282,977	3,280,422
% GC	57,22	57,22
No. of contigs	2	2

*N*50 (bp)	1,161,874	1,161,890
Completeness (%)	97.99	97.99
Contamination (%)	0.71	0.79
BioSample accession no.	SAMN31324765	SAMN31324766
GenBank WGS accession no.	CP109916-CP109917	CP109914-CP109915
SRA accession no. for raw reads	SRR21939256	SRR21939255
